# Respiratory Syncytial Virus Reverses Airway Hyperresponsiveness to Methacholine in Ovalbumin-Sensitized Mice

**DOI:** 10.1371/journal.pone.0046660

**Published:** 2012-10-02

**Authors:** Famke Aeffner, Ian C. Davis

**Affiliations:** Department of Veterinary Biosciences, The Ohio State University, Columbus, Ohio, United States of America; CNRS, France

## Abstract

Each year, approximately 20% of asthmatics in the United States experience acute symptom exacerbations, which commonly result from pulmonary viral infections. The majority of asthma exacerbations in very young children follow infection with respiratory syncytial virus (RSV). However, pathogenic mechanisms underlying induction of asthma exacerbations by RSV are not well understood. We therefore investigated the effect of post-sensitization RSV infection on lung function in ovalbumin (OVA)-sensitized BALB/c mice as a model of RSV asthma exacerbations. OVA sensitization of uninfected female BALB/c mice increased bronchoalveolar lavage fluid (BALF) eosinophil levels and induced airway hyperresponsiveness to the muscarinic agonist methacholine, as measured by the forced-oscillation technique. In contrast, intranasal infection with replication-competent RSV strain A2 for 2–8 days reduced BALF eosinophil counts and reversed airway hyperresponsiveness in a pertussis toxin-sensitive manner. BALF levels of the chemokine keratinocyte cytokine (KC; a murine homolog of interleukin-8) were elevated in OVA-sensitized, RSV-infected mice and reversal of methacholine hyperresponsiveness in these animals was rapidly inhibited by KC neutralization. Hyporesponsiveness could be induced in OVA-sensitized, uninfected mice by recombinant KC or the Gαi agonist melittin. These data suggest that respiratory syncytial virus induces KC-mediated activation of Gαi, resulting in cross-inhibition of Gαq-mediated M_3_-muscarinic receptor signaling and reversal of airway hyperresponsiveness. As in unsensitized mice, KC therefore appears to play a significant role in induction of airway dysfunction by respiratory syncytial virus. Hence, interleukin-8 may be a promising therapeutic target to normalize lung function in both asthmatics and non-asthmatics with bronchiolitis. However, the OVA-sensitized, RSV-infected mouse may not be an appropriate model for investigating the pathogenesis of viral asthma exacerbations.

## Introduction

An estimated 300 million persons worldwide suffer from asthma [1]. Of the 20 million asthmatics in the United States alone, approximately 20% experience an acute deterioration of respiratory symptoms (an asthma exacerbation) in a single year [2]. While most asthma exacerbations are managed in the outpatient setting, more severe episodes may require hospitalization and can even prove fatal [1]. In the U.S., severe asthma exacerbations lead to over 400,000 hospitalizations each year and these hospitalizations constitute about one-third of the total $11.5 billion in annual asthma-related health care expenditures. Viral infections are the most common cause of asthma exacerbations in both children and adults [3]. In children under the age of two years, the majority appear to be caused by respiratory syncytial virus (RSV), although rhinovirus may predominate in older children and adults [4], [5].

In epidemiologic studies, severe RSV bronchiolitis has been associated with development of childhood asthma and episodic bronchospastic bronchitis which may persist into adulthood [6]. Investigators have therefore investigated the impact of infection of neonatal mice with the paramyxoviruses RSV and pneumonia virus of mice on subsequent development of an asthma-like phenotype (induced by ovalbumin [OVA] sensitization and challenge) [7], [8]. Likewise, other studies have examined the effects of RSV infection during OVA challenge on asthma induction in mice [9]–[11]. However, the acute effects of post-sensitization RSV infection on muscarinic receptor signaling in asthma are less well understood. In the current study, we therefore investigated the effects of post-sensitization RSV infection on airway responses to the bronchoconstrictive muscarinic agonist methacholine in the OVA-sensitized mouse, as a model for RSV-induced acute asthma exacerbations. Although we had hypothesized that RSV infection would further increase airway hyperresponsiveness to methacholine in OVA-sensitized animals, we did not find this to be the case. Instead, we found that infection with RSV paradoxically reversed airway hyperresponsiveness to methacholine in a keratinocyte cytokine (KC)-dependent, pertussis toxin-sensitive fashion. This suggests that acute RSV infection modulates muscarinic receptor function in ovalbumin-sensitized mice in a paracrine fashion.

## Materials and Methods

### Ethics Statement

No human subjects or nonhuman primates were involved in this study. All vertebrate animal experiments were approved by The Ohio State University Institutional Animal Care and Use Committee (protocols 2006A0150 and 2009A0083), and were performed in strict accordance with the National Research Council’s Guide for the Care and Use of Laboratory Animals. All surgery was performed under valium/ketamine anesthesia and all efforts were made to minimize suffering.

### Animals

As in our previous studies, 8–10 week-old pathogen-free female BALB/cAnNCr mice (National Cancer Institute, Frederick, MD) were used [12]. Animals were monitored daily for signs of respiratory distress, and were euthanized if this was detected (although this proved to be unnecessary in the current study).

### Preparation of Viral Inocula

Viral stocks were grown in HEp-2 cell monolayers and purified by ultracentrifugation onto a 60% sucrose cushion [13]. Titers were determined by serial dilution and plaque assay in Vero cells under agar [14]. Virus preparations were checked for absence of mycoplasmal and endotoxin contamination [15]. Mock-infected HEp-2 cell supernatant, identically prepared, served as a control for possible effects of cellular components in the inoculum.

### UV Inactivation of RSV

RSV stocks were inactivated by exposure to 1800 mJ of radiation in a Stratalinker UV cross-linker (Stratagene, Cedar Crossing, TX). This protocol eliminates viral infectivity without altering the conformation of viral proteins and mediators [16].

### Ovalbumin Sensitization and Challenge

**Figure 1:**
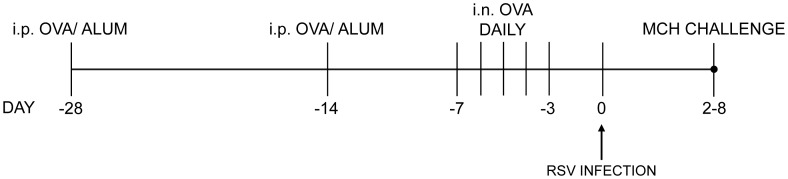
Schematic timeline of the OVA sensitization/challenge and RSV infection protocol. Mice were sensitized by intraperitoneal (i.p.) injection of OVA in alum at days −28 and −14. From −7 to −3 days, mice were challenged daily by intranasal (i.n.) OVA instillation. Animals were infected with RSV 3 days after the last OVA challenge (day 0). Airway responsiveness to methacholine (MCH) was measured at 2–8 days post-infection (d.p.i.).

As described by Pastva *et al.* [17], mice were sensitized on days −28 and −14 by intraperitoneal (i.p.) injection of 50 µg/mouse freshly-prepared grade V chicken OVA (Sigma-Aldrich, St. Louis, MO), suspended in 200 µl/mouse IMJECT alum (Pierce, Rockford, IL). From days −7 to −3, mice were lightly anesthetized daily with isoflurane then challenged by intranasal instillation of 50 µg/mouse OVA in 50 µl sterile saline. Age-matched controls were mock-sensitized with alum only and challenged with saline. A schematic of this protocol is shown in Fig.1.

### Mouse Infection Protocol

3 days after the last OVA challenge (day 0) each mouse was infected intranasally with 10^6^ plaque-forming units (pfu) of RSV strain A2 (in 100 µl) under light isoflurane anesthesia. Mice were placed in lateral recumbency, allowed to recover, then returned to their cages. For all studies, data for each experimental group were derived from a minimum of two independent infections.

### Virus Isolation

Viral replication in mouse lungs was quantified as pfu/g of lung tissue, as described previously [18].

### Measurement of Lung Mechanics

Mechanical properties of the mouse lung were assessed using the forced-oscillation technique, as previously described [12], [19], [20]. Lung function was measured at 2 and 8 days post-infection (d.p.i.) with RSV. These timepoints were selected based on our previous studies showing that RSV maximally impairs airway responses to β-agonists at 2 d.p.i. [18] and undergoes viral clearance by day 8 [12]. Only female mice were used, since male C57BL/6 mice can exhibit exaggerated airway responses to methacholine [21]. Each mouse was anesthetized i.p. with valium (1.75 mg/100 g body weight) followed by ketamine (45 mg/100 g body weight). Once at a surgical plane of anesthesia, the trachea was exposed surgically, a tracheotomy performed, and a trimmed sterile 18-g intravenous catheter inserted caudally into the lumen. Pancuronium was then administered (0.08 µg/kg i.p.). The mouse was mechanically ventilated on a computer-controlled piston ventilator (flexiVent, SciReq; Montreal, Canada), with a tidal volume of 10 ml/kg at a frequency of 200 breaths/minute, against 2–3 cmH_2_O PEEP, as in our previous studies [18]. Following two total lung capacity maneuvers to standardize volume history, pressure and flow data (reflective of airway and tissue dynamics) were collected during a series of standardized volume perturbation maneuvers. These data were used to calculate total lung resistance using the single-compartment model [19].

### Assessment of Airway Responsiveness to Methacholine

Serial dilutions of acetyl β-methacholine (Sigma-Aldrich) in sterile normal saline were prepared fresh daily. To establish baseline total lung resistance, saline was delivered over a 10-second period via an AeroNeb vibrating plate ultrasonic nebulizer, in series with the inspiratory limb of the flexiVent Y-tube. Following a recovery period of 5 seconds, 10 recordings of parameters of lung mechanics were then generated over a 2-minute period. Each recording consisted of a 1.25-second measurement of total lung resistance followed by 2.75 seconds of recovery, then a 3-second quick-prime perturbation with a 5-second recovery period. The mean value of all 10 total lung resistance measurements for that mouse was calculated. Mice were then exposed to increasing doses of methacholine (0.1, 1, 10, 20, and 50 mg/ml). Each methacholine dose was again delivered by nebulization over a 10-second period. 10 recordings of total lung resistance were generated at each methacholine dose, and analyzed as for saline measurements. A 15-second recovery period was interposed between each methacholine dose. Overall group mean values were then calculated for each timepoint or treatment at each methacholine dose.

### Assessment of Acute Modulation of Signaling Pathways by Nebulized Reagents

As indicated in the text, OVA-sensitized mice in some experiments were exposed to agonists or antagonists by nebulization immediately following measurement of baseline total lung resistance. These agents included rat anti-mouse KC monoclonal antibody (50 µg/ml; MAB4531, R & D Systems, Minneapolis, MN), normal rat IgG (50 µg/ml; R & D Systems), recombinant murine KC (50 µg/ml; R & D Systems), and melittin (100 µM; EMD Biosciences, Rockland, MA). As in previous studies, inactivation of recombinant KC was achieved by boiling aliquots for 10 minutes in a waterbath [18]. Inactivation was confirmed by absence of immunoreactivity in a KC ELISA. All reagents were diluted in saline from high-concentration stock solutions immediately prior to use, so that solvent volumes could be minimized to <5 µl/ml. Subsequent to nebulization, responsiveness to methacholine was determined as above.

### Administration of Pertussis Toxin

Pertussis toxin (100 µg/kg in 100 µl saline/mouse, i.p.) was administered 18 hours prior to measuring lung mechanics, as per McGraw *et al.* [22].

### Bronchoalveolar Lavage

Mice were euthanized then tracheotomized as above. The lungs were lavaged *in situ* with 1.0 ml of sterile saline. Bronchoalveolar lavage fluid (BALF) cell viability was determined via trypan blue exclusion and cell types were differentiated on cytospin preps using Wright-Giemsa stain. Cell differentials were determined from at least 200 leukocytes using standard hematological criteria.

### Measurement of Bronchoalveolar Lavage Fluid KC Content

BAL KC content was measured by ELISA (R & D Systems), in accordance with manufacturer’s instructions.

### Statistical Analyses

Descriptive statistics (mean and standard error) were calculated using Instat software (GraphPad, San Diego, CA). Gaussian data distribution was verified by the method of Kolmogorov and Smirnov. Between-group comparisons were made using ANOVA, with a *post hoc* Tukey-Kramer multiple comparison post-test. Mean methacholine dose-response curves for each experimental group were compared using Prism software (GraphPad) by 2-way ANOVA, with *P* values for overall curve comparison across methacholine doses reported. All data are presented as mean ± S.E.M. *P*<0.05 was considered statistically significant.

## Results

### RSV Infection Reduces Bronchoalveolar Lavage Fluid Cell Counts in OVA-sensitized Mice

**Figure 2:**
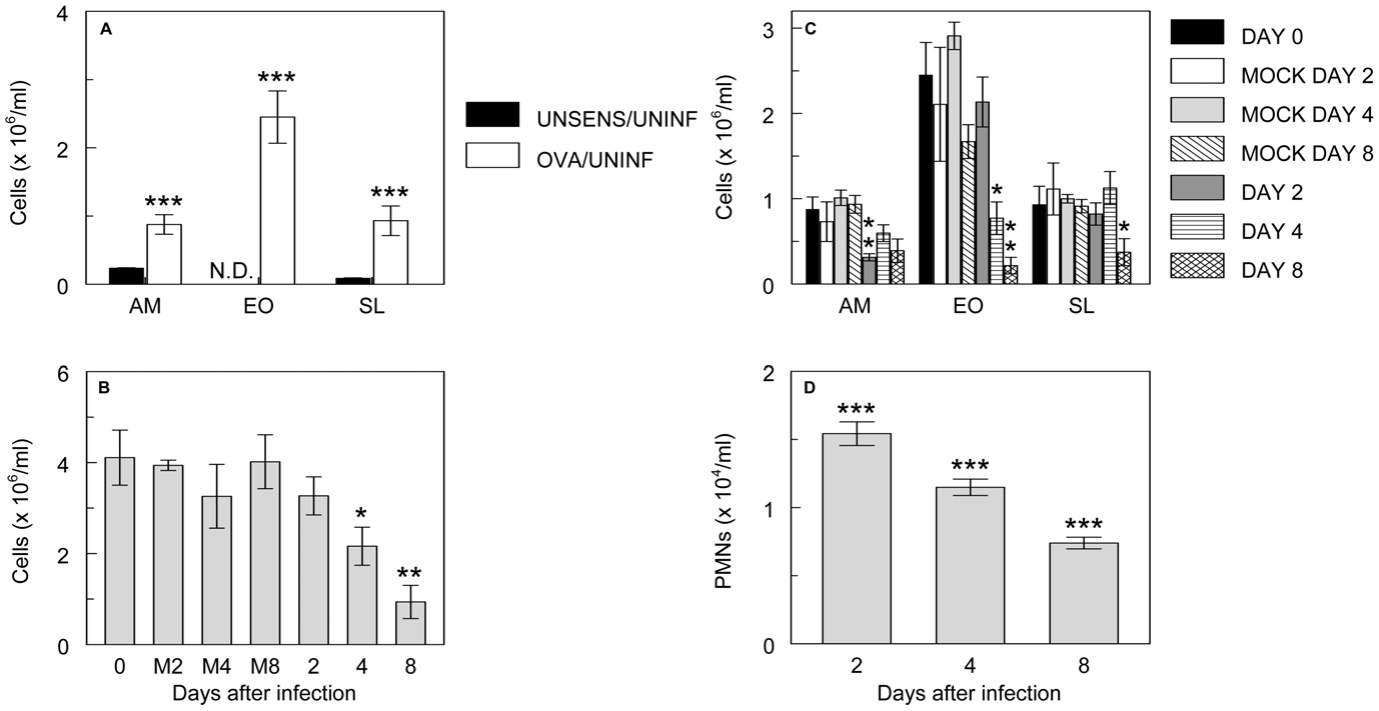
RSV infection reduces bronchoalveolar lavage fluid cell counts in OVA-sensitized mice. Effect of OVA sensitization on (**A**) Alveolar macrophage (AM), eosinophil (EO) and small lymphocyte (SL) counts in uninfected mice (OVA/UNINF; *n* = 8) and unsensitized, uninfected controls (UNSENS/UNINF; *n* = 8); (**B**) Total cell counts in OVA-sensitized mice after mock infection for 2 days (M2; *n* = 5), 4 days (M4; *n* = 4), or 8 days (M8; *n* = 8), and infection with RSV (10^6^ pfu/mouse) for 2 days (*n* = 16), 4 days (*n* = 10), or 8 days (*n* = 6); (**C**) AM, EO, and SL counts after mock infection for 2, 4, or 8 days and infection with RSV for 2–8 days; and (**D**) Neutrophil (PMN) counts after infection with RSV for 2–8 days. No PMNs were detected in bronchoalveolar lavage fluid from uninfected or mock-infected mice at any timepoint (not shown). *P<0.05, **P<0.005, ***P<0.001. N.D.: None detected.

Lungs from control BALB/c mice (mock-sensitized with alum and challenged with saline) were histologically normal. BALF from these animals contained no eosinophils. In contrast, OVA sensitization and challenge of uninfected mice induced airway eosinophilia and moderate goblet cell hyperplasia (data not shown), as reported in prior studies [23]. This was accompanied by a very significant increase in BALF eosinophils, together with elevated alveolar macrophage and lymphocyte counts (Fig. 2A). However, no neutrophils were detected in BALF from either mock-sensitized or OVA-sensitized, uninfected mice.

Intranasal infection of mice with 10^6^ pfu/mouse of sucrose gradient-purified RSV strain A2 (in 100 µl) did not alter goblet cell hyperplasia in OVA-sensitized mice (data not shown), but did trigger a decline in BALF total cell counts from 4–8 d.p.i. (Fig. 2B). This decline, which did not occur in mock-infected mice, primarily resulted from a progressive decrease in BALF eosinophil content (Fig. 2C). A similar reduction in BALF total cell and eosinophil numbers in OVA-sensitized mice was reported by other investigators at 6 and 15 days after RSV infection [9], [11], although the underlying mechanism and biological significance of this effect remains unclear.

Mock infection of OVA-sensitized mice did not induce a neutrophil response. In contrast, RSV infection significantly increased BALF neutrophil counts at 2 d.p.i., although this effect declined over time (Fig. 2D). However, neutrophil infiltration in OVA-sensitized, RSV-infected mice was 100-fold lower than in unsensitized, RSV-infected animals [9], [12], [24].

As in previous studies using this model [9], OVA sensitization did not alter lung RSV replication. Mean lung homogenate viral titers increased from 3.6±0.1 log p.f.u./g lung tissue at 2 d.p.i. (*n* = 5) to a peak of 4.6±0.1 log p.f.u./g at 4 d.p.i. (*n* = 6), but virus was cleared from the lungs by 8 d.p.i. (replication below limits of detection of the assay; *n* = 4). As in our previous studies, we were unable to detect replicating RSV in mice inoculated with UV-inactivated virus at either day 2 or day 8 (*n* = 4 per group). Finally, body weight loss was comparable between unsensitized and OVA-sensitized, RSV-infected mice, and did not differ in severity from our previous studies [15].

#### RSV infection reverses airway hyperresponsiveness to methacholine in OVA-sensitized mice

**Figure 3:**
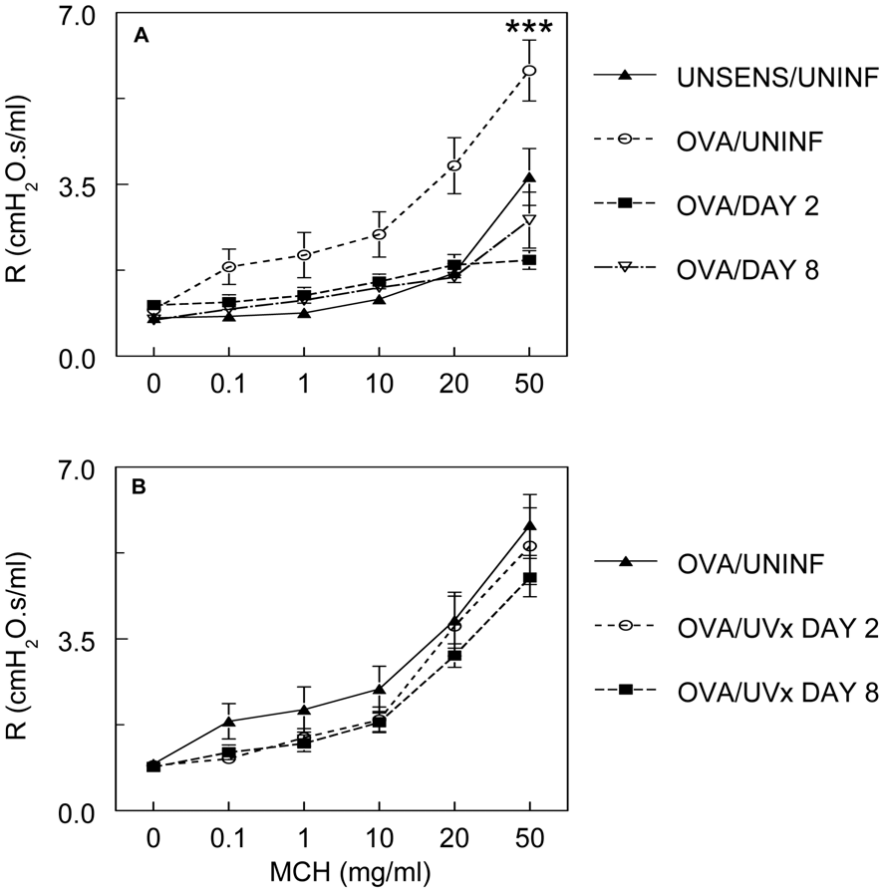
RSV infection reverses airway hyperresponsiveness to methacholine in OVA-sensitized mice. Bronchoconstrictive responses to increasing doses of nebulized methacholine (MCH) in: (**A**) unsensitized, uninfected mice analyzed on day 0 (UNSENS/UNINF; *n* = 6), OVA-sensitized, uninfected mice (OVA/UNINF; *n* = 8), and OVA-sensitized mice infected with RSV (10^6^ pfu/mouse) for 2 days (OVA/DAY 2; *n* = 16), or 8 days (OVA/DAY 8; *n* = 8); (**B**) OVA/UNINF mice and OVA-sensitized mice “infected” with UV-inactivated RSV for 2 days (OVA/UVx DAY 2; *n* = 5) or 8 days (OVA/UVx DAY 8; *n* = 9). ***MCH dose-response curve differs significantly (*P*<0.0005) from UNSENS/UNINF mice.

Following measurement of baseline total lung resistance by the forced-oscillation technique, mice were exposed to increasing doses of methacholine (0.1–50 mg/ml), each of which was administered over 10 seconds by nebulization. Methacholine responsiveness in unsensitized, uninfected BALB/c mice was similar to our previous study [18], and did not differ from alum-sensitized controls (data not shown). As in other studies [11], [20], [25], [26], significant airway hyperresponsiveness to methacholine was present in OVA-sensitized, uninfected animals (Fig. 3A). In contrast, airway hyperresponsiveness to methacholine was absent in OVA-sensitized mice following infection with RSV at both 2 and 8 d.p.i. Indeed, methacholine responsiveness was no different from that of unsensitized, RSV-infected animals at these same timepoints. Importantly, methacholine hyperresponsiveness was present in mice “infected” for 2 or 8 days with RSV which was antigenically-intact but replication-incompetent as a result of exposure to UV light (Fig. 3B) [16]. This latter finding indicates that viral replication is necessary for reversal of methacholine hyperresponsiveness in OVA-sensitized mice, and that this phenomenon does not result solely from administration of large amounts of viral antigen [27]. Moreover, it demonstrates that reversal of methacholine hyperresponsiveness following RSV infection cannot be attributed to any contaminating mediators from the HEp-2 cells in which the virus is cultured, since these would remain functional following UV-crosslinking of viral RNA [16], [18], [28].

#### RSV infection reverses hyperresponsiveness to methacholine in OVA-sensitized mice via a pertussis toxin-sensitive pathway

**Figure 4:**
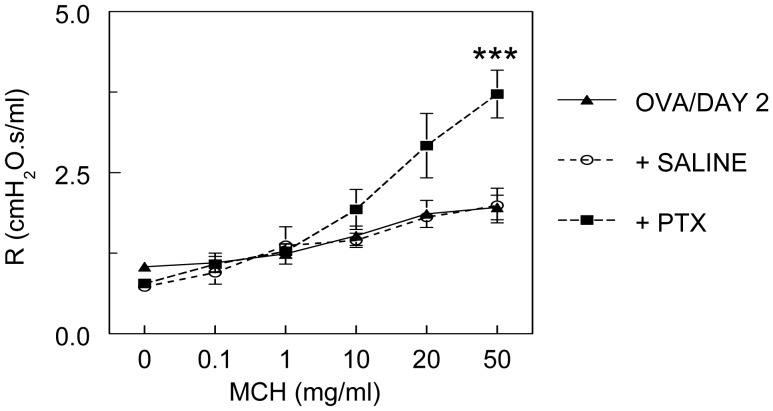
RSV infection reverses hyperresponsiveness to methacholine in OVA-sensitized mice via a pertussis toxin-sensitive pathway. Bronchoconstrictive response to increasing doses of nebulized methacholine (MCH) following pretreatment with saline (100 µl i.p.; *n* = 4) or pertussis toxin (PTX, 100 µg/kg in 100 µl saline i.p.; *n* = 9). ***MCH dose-response curve differs significantly (*P*<0.0005) from OVA/DAY 2 mice (OVA-sensitized mice infected with 10^6^ pfu/mouse RSV A2 for 2 days; *n* = 16).

McGraw *et al.* have reported that unsensitized, uninfected β_2_-adrenergic receptor-knockout FVB mice are hyporesponsive to methacholine [22], [29]. They also showed that pertussis toxin enhanced bronchoconstriction to methacholine, indicating that hyporesponsiveness in their model is mediated by G protein-inhibitory α subunit (Gαi). Similarly, we found that treatment of OVA-sensitized, RSV-infected mice with pertussis toxin (100 µg/kg in 100 µl saline, i.p.), but not the vehicle control (100 µl saline only, i.p.), 18 hours prior to lung function analysis increased responsiveness to methacholine at 2 d.p.i. (Fig. 4). However, pertussis toxin pretreatment was insufficient to induce the airway hyperresponsiveness observed in untreated, OVA-sensitized, uninfected mice.

#### Keratinocyte cytokine released in response to RSV infection reverses hyperresponsiveness to methacholine in OVA-sensitized mice

**Figure 5:**
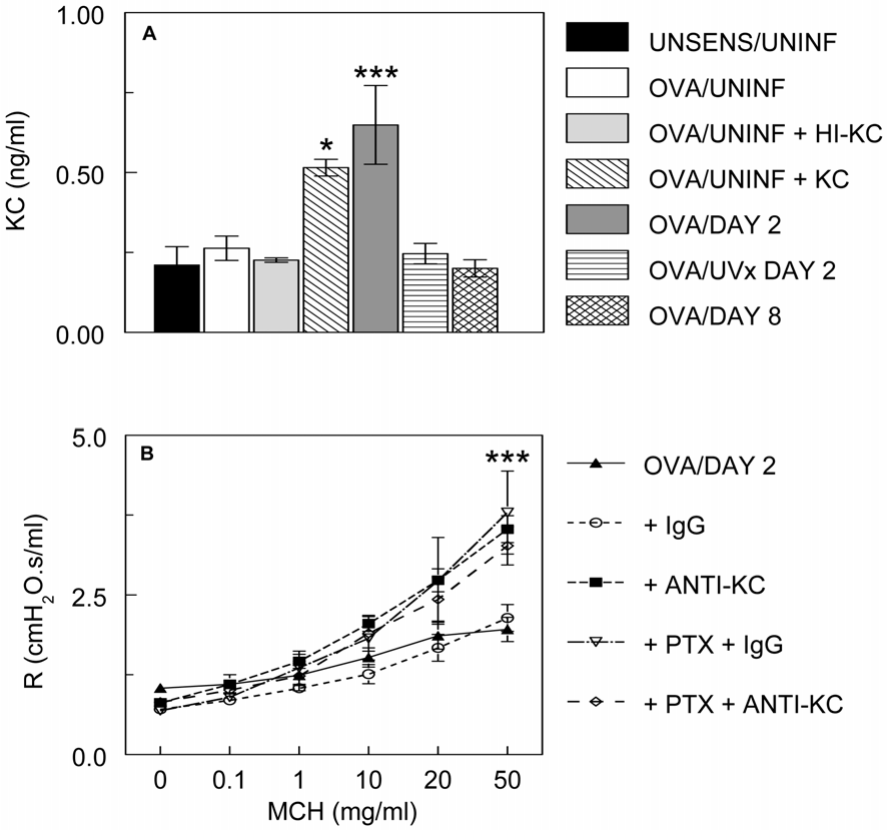
Keratinocyte cytokine released in response to RSV infection reverses hyperresponsiveness to methacholine in OVA-sensitized mice. (**A**) Bronchoalveolar lavage fluid keratinocyte cytokine (KC; ng/ml) levels in unsensitized, uninfected mice (UNSENS/UNINF; *n* = 5), OVA-sensitized, uninfected mice (OVA/UNINF; *n* = 11), OVA-sensitized, uninfected mice treated with 50 µg/ml heat-inactivated recombinant murine KC (OVA/UNINF + HI-KC; *n* = 5), OVA-sensitized, uninfected mice treated with 50 µg/ml recombinant murine KC (OVA/UNINF + KC; *n* = 7), OVA-sensitized mice infected with RSV (10^6^ pfu/mouse) for 2 days (OVA/DAY 2; *n* = 6), OVA-sensitized mice “infected” with UV-inactivated RSV for 2 days (OVA/UVx DAY 2; *n* = 4), and OVA-sensitized mice infected with RSV for 8 days (OVA/DAY 8; *n* = 6). *P<0.05, ***P<0.0005, *vs*. UNSENS/UNINF mice. (**B**) Bronchoconstrictive responses to increasing doses of nebulized methacholine (MCH) in OVA-sensitized, RSV-infected mice following nebulization of normal rat IgG (50 µg/ml; *n* = 5), KC-neutralizing monoclonal antibody (ANTI-KC, 50 µg/ml; *n* = 5), pretreatment with pertussis toxin and IgG (PTX + IgG; *n* = 6), or pretreatment with pertussis toxin and KC neutralizing antibody (PTX + ANTI-KC; *n* = 8). ***MCH dose-response curve differs significantly (*P*<0.0005) from UNSENS/UNINF mice (*n* = 16).

The chemokine KC, which is a murine homolog of interleukin-8 (IL-8), is the predominant inflammatory mediator present in the BAL and lung tissue of mice at early timepoints following RSV infection [12], [30], [31]. We and others have shown that this chemokine can directly alter airway smooth muscle function [18], [32], [33]. Moreover, KC binding to CXCR2 receptors ordinarily induces activation of pertussis toxin-sensitive Gαi [34]. We therefore investigated the role of this chemokine in inducing reversal of methacholine hyperresponsiveness in OVA-sensitized, RSV-infected animals. BALF KC levels did not differ significantly between unsensitized, uninfected mice and OVA-sensitized, uninfected animals (Fig. 5A). However, KC levels were significantly higher in OVA-sensitized mice infected with RSV for 2 days, but not in animals “infected” with UV-inactivated virus for the same time period. Finally, BALF KC content returned to baseline by 8 d.p.i.

Like pertussis toxin, administration of nebulized KC-neutralizing antibody (50 µg/ml) immediately prior to airway function analysis increased responsiveness to methacholine in OVA-sensitized mice infected with RSV for 2 days (Fig. 5B). An equivalent amount of nonspecific rat IgG had no such effect. Again, like pertussis toxin, anti-KC treatment did not induce airway hyperresponsiveness. Moreover, the effects of pertussis toxin and KC blockade on methacholine responsiveness were neither additive nor synergistic, suggesting that both agents act upon the same pathway.

#### Keratinocyte cytokine and Gαi activation are both sufficient to reverse methacholine hyperresponsiveness in OVA-sensitized, uninfected mice

**Figure 6:**
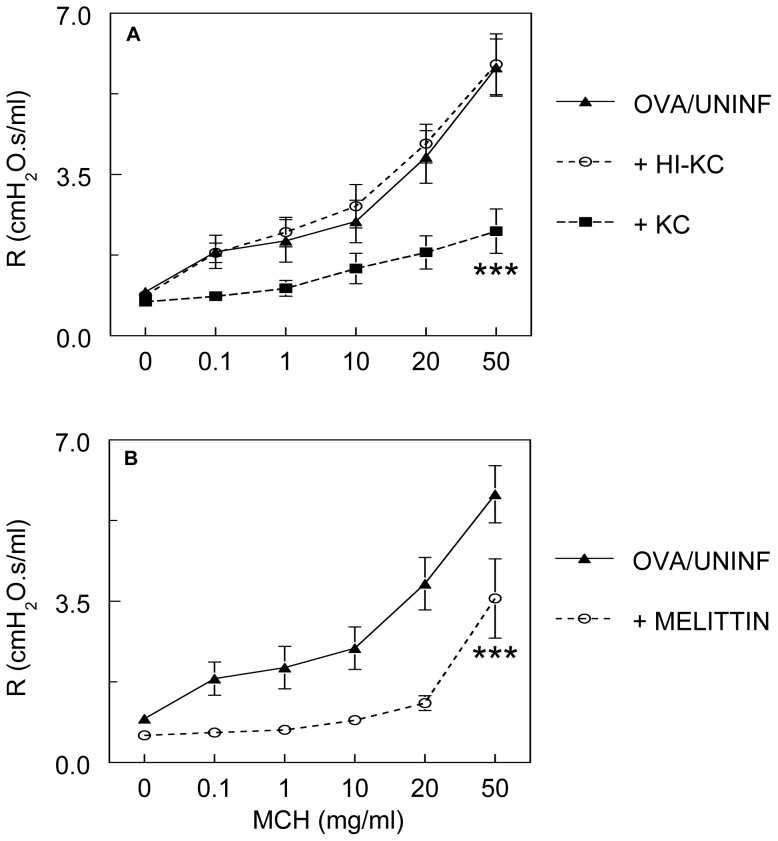
Keratinocyte cytokine exposure and Gαi activation are both sufficient to reverse methacholine hyperresponsiveness in OVA-sensitized, uninfected mice. Bronchoconstrictive responses to increasing doses of nebulized methacholine (MCH) following nebulization of: (**A**) heat-inactivated recombinant murine keratinocyte cytokine (HI-rmKC, 50 µg/ml; *n* = 6) or recombinant murine keratinocyte cytokine (rmKC, 50 µg/ml; *n* = 7); and (**B**) Melittin (100 µM; *n* = 7). ***MCH dose-response curve differs significantly (*P*<0.0005) from OVA/UNINF mice (*n* = 8).

The above data imply that RSV-induced reversal of methacholine hyperresponsiveness should be reproducible in uninfected mice by activation of KC receptors or downstream pertussis toxin-sensitive Gαi-mediated signaling. We therefore exposed OVA-sensitized, uninfected mice to recombinant murine KC (50 µg/ml) or the Gαi inducer melittin (100 µM) by nebulization immediately prior to airway function analysis. Following nebulization of recombinant KC, mean BALF KC content increased to a level comparable to that of OVA-sensitized, RSV-infected mice at 2 d.p.i. (see Fig. 5A), indicating that addition of 50 µg/ml KC by nebulization recapitulates the effect of RSV infection on this chemokine. Heat-inactivated recombinant KC did not increase BALF KC content. This indicates that heat treatment destroys the antigenicity of KC and is therefore likely to render it biologically inert. Both KC and melittin significantly and comparably reduced airway responsiveness to methacholine relative to untreated controls (Figs. 6A and 6B, respectively). The effect of KC was lost following heat-inactivation.

## Discussion

The majority of asthma exacerbations in very young children result from RSV infection [3], [5]. However, pathogenic mechanisms underlying induction of asthma exacerbations by RSV are not well understood. We therefore investigated the effect of post-sensitization RSV infection on lung function in OVA-sensitized BALB/c mice as a model of RSV asthma exacerbations. As in previous studies [11], [20], [25], [26], we found that OVA sensitization induced airway hyperresponsiveness to methacholine in uninfected mice. Unexpectedly, however, post-sensitization infection with replication-competent RSV for 2–8 days reversed this effect. In addition, reversal of OVA-induced airway hyperresponsiveness was mediated by the chemokine KC in a pertussis toxin-sensitive manner. These findings indicate that RSV modulates Gαi signaling in OVA-sensitized mice, resulting in paradoxical effects on airway responsiveness to methacholine. However, these paradoxical effects also suggest that the OVA-sensitized, RSV-infected mouse may not be an appropriate model for investigating the pathogenesis of viral asthma exacerbations.

In unsensitized, uninfected mice, methacholine binds to M_3_-subtype muscarinic receptors, resulting in release of Gαq and downstream activation of phospholipase C. Phospholipase C then activates protein kinase C and increases intracellular Ca^++^, leading to bronchoconstriction. Following sensitization with OVA, uninfected mice became hyperresponsive to methacholine, but this effect was reversed by RSV infection. Reversal of methacholine hyperresponsiveness has not previously been reported in mice infected with RSV post-sensitization with OVA. Indeed, prior studies have provided somewhat conflicting results regarding the impact of RSV on airway responses to methacholine, although many have reported that RSV enhances methacholine responsiveness in OVA-sensitized mice [7], [35], [36]. We hypothesize that such variables as the route of methacholine administration, the timing of RSV infection relative to that of OVA sensitization, the virus strain used, and the post-infection timepoints analyzed may account for differences between our results and those of previous investigators. For example, Peebles *et al.* found no difference in airway methacholine responsiveness between OVA-sensitized, uninfected and OVA-sensitized, RSV-infected mice at day 8, although RSV significantly enhanced airway hyperresponsiveness to intravenous methacholine at day 15 [11]. Moreover, pre-sensitization RSV infection resulted in hyporesponsiveness to intravenous methacholine, but post-sensitization infection induced airway hyperresponsiveness [37]. Makela *et al.* also reported airway hyperresponsiveness at day 6 in OVA-sensitized, RSV Long strain-infected C57BL/6 mice [26]. However, C57BL/6 mice differ significantly from BALB/c mice in methacholine responsiveness [38]. Moreover, unlike the A2 strain, the Long strain of RSV also induces airway hyperresponsiveness in unsensitized animals [11], [18], [39], [40]. Finally, we found that reversal of methacholine hyperresponsiveness was most significant at day 2 following RSV infection. This timepoint was not examined in comparable prior studies.

The chemokine KC is the predominant proinflammatory mediator in the lungs of unsensitized, RSV-infected mice at early post-infection timepoints [18], [28], but is not induced in response to challenge with UV-inactivated virus [9]. In previous studies we demonstrated that infection with replication-competent RSV induces both bronchoalveolar and airway epithelial insensitivity to β-agonists in a KC-dependent fashion [18], [28]. Similarly, we found in the current study that the increase in lung KC levels induced by infection of OVA-sensitized mice with replication-competent RSV was sufficient to reverse methacholine hyperresponsiveness. Likewise, hyperresponsiveness to methacholine in OVA-sensitized, uninfected mice could be reversed by exposure to a physiologically-relevant dose of recombinant KC alone. Methacholine hyporesponsiveness has previously been described in mice overexpressing Gαi, and the IL-13-knockout mouse asthma model [22], [29]. Based on their findings in mice overexpressing Gαi, McGraw *et al.* postulated that hyporesponsiveness to methacholine may result from crosstalk between Gαi and the Gαq pathway at the level of phospholipase C [22]. In this model, they proposed that increased Gαi activity reduces airway reactivity by blocking the activation of phospholipase C, and may therefore play a bronchoprotective role in asthma. Our findings are consistent with these prior studies, since we found that Gαi activation by KC or melittin was sufficient to reduce airway reactivity. However, because inhibition of phospholipase C with U-73122, inhibition of protein kinase C with BIM I, and chelation of intracellular Ca^++^ with BAPTA-AM all inhibited airway hyperresponsiveness in OVA-sensitized, uninfected mice (data not shown), it was not possible to determine effects of RSV on this downstream pathway directly.

**Figure 7:**
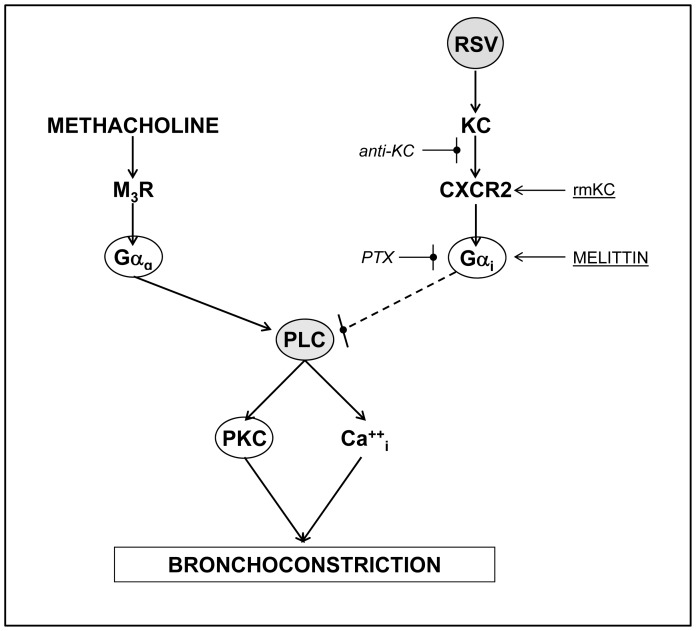
Proposed mechanism for altered airway responsiveness to methacholine in ovalbumin-sensitized mice following RSV infection. In unsensitized or OVA-sensitized, uninfected mice, methacholine binds to M_3_-subtype muscarinic receptors (M_3_R), resulting in release of Gαq and downstream activation of phospholipase C (PLC). PLC then activates protein kinase C (PKC) and increases intracellular Ca^++^ (Ca^++^_i_), leading to bronchoconstriction. RSV infection of respiratory epithelial cells in OVA-sensitized mice induces release of keratinocyte cytokine (KC), which binds to epithelial CXCR2 receptors in either an autocrine or paracrine fashion. KC receptor binding can be replicated in OVA-sensitized, uninfected mice by recombinant murine KC (rmKC), but is blocked by a neutralizing antibody to KC (*anti-KC*). Activation of CXCR2 liberates pertussis toxin (*PTX*)-sensitive Gαi, resulting in reversal of hyperresponsiveness to methacholine. Gαi can also be directly activated by melittin. As in previous studies [22], [29], reversal of airway hyperresponsiveness may be a consequence of inhibition of the phospholipase C (PLC)/protein kinase C (PKC) pathway by Gαi, although we did not formally demonstrate this in the current study. Experimental agonists are shown underlined. Experimental antagonists are shown in italics. Broken lines indicate postulated mechanisms that were not formally demonstrated in this study.

Together with previous studies, our data suggest that KC reverses hyperresponsiveness to methacholine in OVA-sensitized, RSV-infected mice by the following mechanism (Fig. 7): KC binds to CXCR2 receptors, which ordinarily couple to pertussis toxin-sensitive Gαi [34]. Gαi activation then reduces responsiveness to methacholine. We can infer from previous studies by McGraw *et al*. that reduced methacholine responsiveness most probably results from inhibition of the phospholipase C signaling pathway by Gαi [22], [29], [41]. Importantly, RSV has not previously been shown to induce Gαi activation in the murine lung. However, it is important to note that our proposed mechanism does not account for all of the functional effects of RSV infection in OVA-sensitized animals. For example, pertussis toxin treatment and KC blockade could not restore the asthma-like airway hyperresponsiveness to methacholine which was present in OVA-sensitized, uninfected mice. Finally, we cannot exclude the possibility that RSV increases airway Gαi expression in OVA-sensitized mice, as was reported by McGraw *et al.* in uninfected animals [22]. However, the ability of nebulized recombinant KC to reverse methacholine hyperresponsiveness within 20 minutes in OVA-sensitized, uninfected mice would suggest that this mechanism is unlikely.

One limitation of the current study is that mice are only a semi-permissive host for RSV. Indeed, some investigators have proposed that the mild clinical disease resulting from RSV inoculation in mice is replication-independent and in fact reflects challenge with and clearance of a large quantity of viral antigens [27]. RSV has been shown to activate toll-like receptors (TLRs)-2, -3, and -4 as well as protein kinase R and RIG-I [42]. As in our previous studies in unsensitized mice [18], we found that UV-inactivated RSV did not induce airway hyporesponsiveness to methacholine in OVA-sensitized animals. Since UV treatment is unlikely to inactivate ligands of TLR-2 or TLR-4, which are expressed on the host cell surface, this finding suggests that these receptors are not involved in RSV-induced methacholine hyporesponsiveness. Likewise, since ligands for TLR-3, protein kinase R, and RIG-I (double-stranded RNA intermediates) are only generated during viral replication our data indicate that induction of methacholine hyporesponsiveness is replication-dependent. Nevertheless, given the semi-permissive nature of the mouse for RSV replication, induction of airway hyporesponsiveness in OVA-sensitized animals may not fully reflect the effects of RSV in human asthmatics. Hence, both the inherent limitations of the RSV mouse model and the paradoxical effects of this virus on airway function in previously-sensitized mice indicate that the OVA-sensitized, RSV-infected mouse may not be appropriate for investigating the pathogenesis of viral asthma exacerbations. Although less widely-used, other paramyxoviruses such as Sendai virus and pneumonia virus of mice cause more severe disease in this species [27]. It is therefore possible that infection of OVA-sensitized mice with these pathogens may better model human viral asthma exacerbations. Both viruses have been shown to promote airway hyperresponsiveness when mice are infected either prior to or during OVA sensitization [8], [43]. Unfortunately, however, effects on airway function of post-sensitization infection with either Sendai virus or pneumonia virus have not been reported to our knowledge.

In conclusion, we found that RSV infection of OVA-sensitized mice reversed airway hyperresponsiveness to the bronchoconstrictor methacholine. Reversal of airway hyperresponsiveness was induced by the chemokine KC, and could be replicated by direct activation of pertussis toxin-sensitive Gαi. This suggests that reversal results from Gαi-mediated cross-inhibition of phospholipase C, which is normally activated by Gαq in response to binding of methacholine to M_3_-subtype muscarinic receptors. Our data indicate that KC released in response to RSV infection triggers a previously unrecognized increase in Gαi activity in OVA-sensitized mice, which results in significant derangement of airway responses to muscarinic agonists. The effect of RSV on methacholine responsiveness in the OVA-sensitized mouse is rather paradoxical, which suggests that this model may be of limited value for studies of viral asthma exacerbations. Nevertheless, when viewed in the context of our previous findings [18], [28], these studies reinforce the potential importance of IL-8 as a therapeutic target following RSV infection.
